# Fluorescence Lifetime Imaging and Spectroscopic Co-Validation for Protoporphyrin IX-Guided Tumor Visualization in Neurosurgery

**DOI:** 10.3389/fonc.2021.741303

**Published:** 2021-09-14

**Authors:** David Reichert, Mikael T. Erkkilae, Johanna Gesperger, Lisa I. Wadiura, Alexandra Lang, Thomas Roetzer, Adelheid Woehrer, Marco Andreana, Angelika Unterhuber, Marco Wilzbach, Christoph Hauger, Wolfgang Drexler, Barbara Kiesel, Georg Widhalm, Rainer A. Leitgeb

**Affiliations:** ^1^Center for Medical Physics and Biomedical Engineering, Medical University of Vienna, Vienna, Austria; ^2^Christian Doppler Laboratory OPTRAMED, Medical University of Vienna, Vienna, Austria; ^3^Division of Neuropathology and Neurochemistry, Department of Neurology, Medical University of Vienna, Vienna, Austria; ^4^Department of Neurosurgery, Medical University of Vienna, Vienna, Austria; ^5^Advanced Development Microsurgery, Carl Zeiss Meditec AG, Oberkochen, Germany

**Keywords:** fluorescence-guided surgery, fluorescence lifetime imaging (FLIM), fluorescence spectroscopy, protoporphyrin IX, surgical microscope

## Abstract

Maximal safe resection is a key strategy for improving patient prognosis in the management of brain tumors. Intraoperative fluorescence guidance has emerged as a standard in the surgery of high-grade gliomas. The administration of 5-aminolevulinic acid prior to surgery induces tumor-specific accumulation of protoporphyrin IX, which emits red fluorescence under blue-light illumination. The technology, however, is substantially limited for low-grade gliomas and weakly tumor-infiltrated brain, where low protoporphyrin IX concentrations are outweighed by tissue autofluorescence. In this context, fluorescence lifetime imaging has shown promise to distinguish spectrally overlapping fluorophores. We integrated frequency-domain fluorescence lifetime imaging in a surgical microscope and combined it with spatially registered fluorescence spectroscopy, which can be considered a research benchmark for sensitive protoporphyrin IX detection. Fluorescence lifetime maps and spectra were acquired for a representative set of fresh *ex-vivo* brain tumor specimens (low-grade gliomas n = 15, high-grade gliomas n = 80, meningiomas n = 41, and metastases n = 35). Combining the fluorescence lifetime with fluorescence spectra unveiled how weak protoporphyrin IX accumulations increased the lifetime respective to tissue autofluorescence. Infiltration zones (4.1ns ± 1.8ns, *p = 0.017*) and core tumor areas (4.8ns ± 1.3ns, *p = 0.040*) of low-grade gliomas were significantly distinguishable from non-pathologic tissue (1.6ns ± 0.5ns). Similarly, fluorescence lifetimes for infiltrated and reactive tissue as well as necrotic and core tumor areas were increased for high-grade gliomas and metastasis. Meningioma tumor specimens showed strongly increased lifetimes (12.2ns ± 2.5ns, *p = 0.005*). Our results emphasize the potential of fluorescence lifetime imaging to optimize maximal safe resection in brain tumors in future and highlight its potential toward clinical translation.

## Introduction

A large variety of different tumors might occur with the human brain. With an incidence rate of 7.1 and 16.7 per 100,000, around 25,000 primary malignant and 59,000 non-malignant brain and central nervous system tumors were expected to be diagnosed in the United States in 2020 (1). According to the current World Health Organization (WHO) classification of primary brain tumors, gliomas are classified as WHO grades II, III, and IV. Considering recent epidemiologic data, glioblastomas WHO grade IV account for 50 % of malignant brain tumors with a median survival of about 8 months (1). The median survival of lower-grade gliomas (LGG) including WHO grades II and III like diffuse astrocytomas and oligodendrogliomas ranges from 3 to 10 years, respectively (1). LGG account for approximately 20% of all primary brain tumors in adults (2). Meningiomas (MNG) represent the most common primary brain tumor accounting for approximately 38% of cases (1). Such tumors are divided into WHO grades I, II, and III, whereas MNG WHO grade I represents by far the most common tumor with a very good prognosis. Secondary brain tumors (metastases; MET) metastasizing frequently from lung, melanoma, renal, breast, or other cancers are common and characterized by poor patient prognosis (3).

Surgical resection is the treatment of choice in the initial management of gliomas and other brain tumor entities. The extent of resection (EoR) was found to be among the most important prognostic factors in both high-grade gliomas (HGG) and LGG, improving the overall and progression-free survival (4–7). Postoperative adjuvant radio- and/or chemotherapy is initiated dependent on the histopathological tumor diagnosis (8). Due to the proximity of brain tumors to eloquent neurological areas, precise surgical guidance in brain tumors is crucial. The infiltrative nature of glioma is a special challenge for the neurosurgeon. Further, the infiltration of brain tissue favors tumor recurrence, which together with the interdisciplinary treatment makes brain tumors the cancer group with the highest initial cost of care (9). In GBM, solid tumor tissue can be outlined by gadolinium contrast enhancement on preoperative magnetic resonance imaging (MRI) and used for intraoperative neuronavigation. Intraoperative MRI can be employed to update preoperative data and has shown to significantly improve the EoR (10). However, the technology as of now is expensive and not widely available. Novel surgical microscopes provide the possibility to integrate navigation data using preoperative imaging data in combination with intraoperative tumor visualization. In this context, optical imaging modalities are of particular interest. Protoporphyrin IX (PpIX) fluorescence guidance has been approved for medical use in both Europe and the United States and is well established for photodynamic diagnosis (PDD) of malignant glioma (11–13). In PDD, presurgical administration of 5-aminolevulinic acid (5-ALA) induces tumor-specific accumulation of the photosensitizer PpIX (14–16), which emits red fluorescence upon blue light illumination. The efficacy of PpIX for the resection of malignant glioma was first demonstrated in a multicenter phase III study (11). A multitude of subsequent studies has consolidated evidence for PDD to increase the EoR and to delineate tumorous tissue beyond gadolinium contrast enhancement especially in HGG (7, 12, 13, 17). Most LGG and weakly infiltrated tumor areas of HGG, however, lack visual fluorescence during surgical resection. Studies investigating on PpIX fluorescence guidance in LGG report visible fluorescence in about 0% to 20% of patients (17–20). Cases with visible fluorescence in radiologically suspected LGG were for the most part attributed to anaplastic foci, circumscribed intratumoral areas with a higher probability for malignant transformation (17, 20). This together with the frequent infiltrative growth makes complete resection of the fluid-attenuated inversion recovery (FLAIR)/T2 hyperintense lesion in LGG particularly challenging. Both optical-sectioning microscopy on a cellular level, scanning fiber endoscopes and spectroscopic PpIX quantification, however, had shown measurable accumulations of PpIX in LGG or at the boundaries of infiltrative gliomas which both lacked visible fluorescence during surgery (7, 21–24). As the weak PpIX fluorescence is outweighed by a dominating background of tissue autofluorescence in these cases, sensitive PpIX detection inherently requires distinction of PpIX from spectrally overlapping fluorophores. This essentially corresponds to the specificity of PpIX detection.

An alternate approach to measuring spectrally resolved fluorescence is time-resolved fluorescence measurements. The fluorescence lifetime is the average intramolecular time delay between excitation of a fluorophore and the emission of fluorescence. It is determined by the intrinsic molecular properties and the direct molecular environment. At the same time, it is independent of factors as excitation intensity or the method of measurement, which can be based on both time- and frequency-domain principles. The interested reader is referred to Marcu and Hartl (25) as well as Berezin and Achilefu (26). Fluorescence lifetime imaging (FLIM) of endogenous fluorophores like nicotinamide adenine dinucleotide (NADH) and flavin adenine dinucleotide (FAD) has been used in several studies to delineate tumor boundaries (25, 27). The lifetimes of these metabolic coenzymes are linked to the metabolic state of the respective tissue (28, 29). FLIM for macroscopic detection of PpIX, however, was proposed only recently (30). While the fluorescence lifetime of PpIX in organic solution (31) was measured to be 16.4ns, autofluorescence lifetimes of physiological parenchyma in murine and human brain were in the range of about 0.8 to 2 ns (detection > 580 nm) (32, 33). This is in good agreement with lifetimes of the individual endogenous fluorophores contributing to the overall autofluorescence in this spectral band (34).

Based on our previous results (32, 35), we hypothesize that weak PpIX concentrations as found in LGG and weakly infiltrated tumor tissue of HGG increase the fluorescence lifetime respective to non-pathological brain parenchyma when measured with frequency-domain (FD)-FLIM. To investigate the extent to which PpIX fluorescence can be distinguished from a dominating autofluorescence background, we developed a multimodal surgical microscope. We combined FD-FLIM with spatially registered fluorescence spectroscopy, which can be considered a research benchmark for sensitive PpIX detection (7, 36). It is of note that while these studies accounted for optical tissue properties to quantify PpIX concentrations in tissue, our work relies on the raw fluorescence signal and employs the relative PpIX to autofluorescence signal contribution as a metric. To substantiate our hypothesis, we acquired a representative data set on freshly resected LGG, HGG, MNG, and MET. Next to emphasizing the capacity of FD-FLIM to delineate weak PpIX fluorescence, our second objective was to engineer the technology toward coherence with surgical workflows. We propose an updated fiber-optic-based implementation and demonstrate how both high-resolution tissue structure and FD-FLIM images can be delivered to the surgeon in real time through long working-distance surgical microscopes. This study emphasizes the potential of FLIM to improve maximal safe resection in brain tumor neurosurgery and highlights the feasibility of clinical translation.

## Materials and Methods

### Multimodal FD-FLIM and Spectroscopy Setup

**Figure 1:**
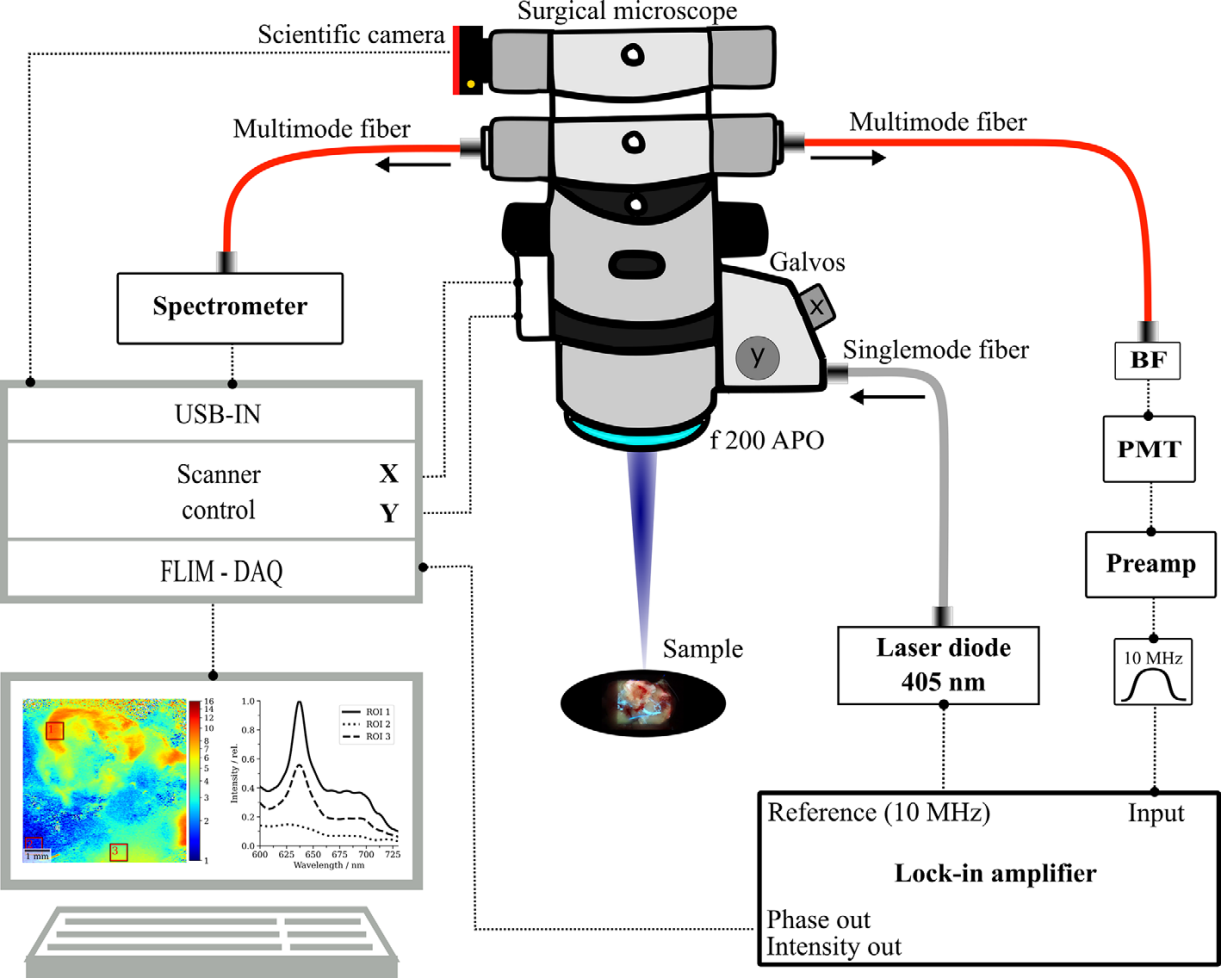
Schematic sketch of the multimodal surgical microscope with integrated fluorescence lifetime imaging and spectroscopy channels. BF, bandpass filter; PMT, photomultiplier tube; DAQ, data acquisition system, f 200 APO, apochromatic objective.

With the current setup (**Figure 1**), we expand on our previously published FD-FLIM system, integrated into a long working-distance (200 mm) surgical microscope (OPMI VISU 200, Carl Zeiss Meditec AG, Jena, Germany). Concisely, the FLIM engine consists of raster-scanning a modulated 405-nm laser (*ƒ_mod_* = 10MHz) for excitation and demodulating the emitted fluorescence signal *via* phase-sensitive detection. Upon correction of time-of-flight and electronic phase delays, the fluorescence lifetime *τ* is related to the measured phase by $\tau =\frac{\tan(\Phi )}{2\pi f_{mod}}$ (37). For a detailed description of the measurement setup and equipment, please refer to our previous work (38). A figure of the modified surgical microscope is depicted in **Supplementary Figure 1**. In the current iteration, we opted to couple the fluorescence into a 1,500-µm core multimode fiber (FP1500URT, 0.5NA, Thorlabs Inc., Newton, NJ, USA), rather than attaching the photomultiplier tube directly to the microscope. PpIX was detected in the band from 590 to 740 nm (665/150 BrightLine HC Semrock, Rochester, NY, USA). A single fluorescence lifetime frame with a lateral resolution of 25 µm (256 × 256 pixels) was acquired in 16 s. The excitation power on the sample was 6 mW and the fluorescence collection numerical aperture for a single stereoscopic path was 0.04. The 50/50 splitter in the right stereoscopic path was exchanged by a mirror to maximize fluorescence throughput. Further, the pixel dwell time and the integration time of the lock-in amplifier were set to 250 and 200 µs, respectively, to allow for sufficient settling of the low-pass filter’s output. Fluorescence emission in the left stereoscopic path was split (50/50) and then coupled into a 1,500-µm core multimode fiber before detection by a spectrometer (CCS100/M, Thorlabs Inc.). Spectra were acquired only for selected regions of interest (ROIs) (size 600 µm^2^) on the samples. To do so, arbitrary X/Y pixel coordinates were selected on a raster-scanned lifetime image and were then rendered into the corresponding scanner angles by a custom MATLAB script. The galvanometer mirrors then steered the laser on the respective ROIs. As spectra were acquired through a surgical microscope, integration time had to be increased up to 50 s to collect sufficient photons for weakly fluorescent tissue. Note that acquiring spatially correlated FD-FLIM and spectroscopic data would not have been possible with our previously published wide-field FD-FLIM setup (30), which is why we opted for the raster-scanning approach (38) within this work. We applied background correction and Gaussian filtering and could thereby recover spectra even for weak autofluorescence. Before the ocular, a 1.6-megapixel complementary metal-oxide-semiconductor camera (CS165CU1/M, Thorlabs Inc.) was attached to a second 50/50 splitter. Thus, images comparable to the surgeon’s white-light tissue view could be acquired with the light source of the surgical microscope turned on. To acquire fluorescence images, the light source was turned off and the laser was scanned rapidly across the tissue. Camera integration time was then set to a long exposure time (2 s for most specimens). For both the camera and the spectrometer channels, light from the laser diode was blocked by a 430-nm longpass filter (430/LP BrightLine HC, Semrock).

### Patient Cohort and Specimen Handling

Data were acquired in the laboratory in an *ex vivo* study on human brain tumor specimens from routine cytoreductive surgery. Diagnostic MRI was performed prior to surgery, with at least T1-weighted sequences with and without contrast media and T2-weighted sequences. Depending on tumor localization, additional sequences such as functional MRI and/or diffusion tensor imaging were conducted. Surgical resection was assisted by neuronavigation, white-light microscopy, and fluorescence guidance with 5-ALA administration 3 h before anesthesia. A small subset of patients without 5-ALA administration was included in the study. After resection, specimens were kept in a physiological environment (artificial cerebrospinal fluid, Landesapotheke Salzburg, 19C11S02) and imaged within 1 h. Histopathological diagnosis and representative H&E-stained slides were provided by experienced neuropathologists according to the WHO classification of CNS tumors from 2016.

In total, 15 LGG, 80 HGG, 41 MNG, and 35 MET specimens were included in the study. **Supplementary Tables 1–6** as well as **Supplementary Figure 4** provide a comprehensive overview of all specimens including WHO grade and patient-specific data as age and gender for the study cohort. Tumor entities were further categorized into core tumor (TUM), diffusely infiltrated brain (INF), necrosis (NEC), and reactive parenchyma (REA), according to the histopathological workup. Reactive tissue refers to non-infiltrated tissue with reactive alterations such as inflammatory responses. As non-pathological tissue usually is not resected, we only imaged one control sample resected on the access route to a tumor. For statistical purposes, we included two non-pathological specimens from our previous work (32, 35), resulting in three specimens in the non-pathological control (CTL) group. The study was approved by the ethics committee (EK419/2008 – Amendment 04/2018), and informed and written consent of patients was obtained.

### Processing Routines and Statistical Analysis

Post-processing routines for reconstructing the fluorescence lifetime and intensity images from the lock-in amplifier’s demodulated phase Θ and amplitude R were implemented in Jupyter Notebook. Scientific computations, statistical analysis, and data visualization were performed with Python’s (RRID: SCR_008394) SciPy, Seaborn, and Matplotlib libraries. For spectra acquired on selected ROIs of the FLIM images, the relative PpIX signal contribution at the main emission peak (635 nm) was defined as a metric. A linear regression model was fit to the data points for wavelengths between 600 and 610 nm and 720 and 730 nm. Photon counts above the resulting fit were contributed to PpIX (S_PpIX_) and below to tissue autofluorescence (S_Autofl._). The relative PpIX signal contribution (RSC_PpIX_) was calculated as $RSC_{PpIX}=\frac{S_{PpIX}}{S_{Autofl.}+S_{PpIX}}$. About three to four spectra were measured for a total of 99 samples which resulted in 331 data points. Spectra were labeled according to the fluorescence status observed by the operating surgeon (visible/no visible fluorescence). For samples with inhomogeneous intraoperative fluorescence, the fluorescence status of the respective ROIs was additionally judged during imaging. A fifth-order polynomial model was fit to the data to approximate the relation between PpIX signal contribution and fluorescence lifetime. Confidence intervals (CI) (39) were calculated based on a two-sided 95% t-statistic, indicating the probability of the interval to contain the mean response of new data points.


$$CI=t_{DOF}^{0.975}\cdot \hat{\sigma }\cdot \sqrt{\frac{1}{n}+\frac{{\left(x_{0}-\bar{x}\right)}^{2}}{\Sigma_{i=1}^{n}{\left(x_{i}-\bar{x}\right)}^{2}}}$$

The $t_{DOF}^{0.975}$- quantile was determined as a function of the degrees of freedom (DOF), and a two-sided 95% level of significance and $\bar{x}$ was the mean relative PpIX signal contribution. DOF were calculated as the sample size minus the number of parameters (6 for a fifth-order polynomial fit). The standard deviation of the error term $\hat{\sigma }$ was calculated as


$$\hat{\sigma }=\sqrt{\sum_{i=1}^{n}\frac{{\left(y_{i}-y_{model,i}\right)}^{2}}{DOF}}$$

with *y_i_* – *y_model,i_* being the residual error between a model estimation and the respective measured lifetime. Similarly, prediction intervals (PI) were determined, giving a 95% probability for a new observation to be within the PI.


$$PI=t_{DOF}^{0.975}\cdot \hat{\sigma }\cdot \sqrt{1+\frac{1}{n}+\frac{{\left(x_{0}-\bar{x}\right)}^{2}}{\Sigma_{i=1}^{n}{\left(x_{i}-\bar{x}\right)}^{2}}}$$

CI and PI were then added to the model predictions as *y_model,i_* ± *CI* and *y_model,i_* ± *PI* to form the respective bands.

Descriptive statistics on lifetime data included the mean and standard deviation as well as the median and first and third quartiles. The distribution of those values was visualized in violin plots according to the respective histopathological classification. To assure equal contribution of all specimens, the number of evaluated pixels per sample was normalized to the smallest specimen. This resulted in 21,733 randomly selected pixels per specimen being included in the analysis.

For inferential statistics on differences between the tumor entities and a control group containing non-pathological tissue, we employed a non-parametric Mann–Whitney U test. This decision was based on a low number of specimens within some subgroups (TUM, INF, NEC, REA) of tumor entities. Differences in the distributions among subgroups were ruled out with the Kolmogorov–Smirnov test after data normalization. This is a prerequisite when comparing medians with the Mann–Whitney U test. We considered differences between groups to be significant if p < 0.05, with the alternate hypothesis stating that the median fluorescence lifetime was significantly greater than in the non-pathological control group.

For FD-FLIM representation in images, we color-coded the lifetime with the python jet color map. Scaling this color map with the binary logarithm allowed for contrasting the dynamic range from tissue autofluorescence and LGG to strongly fluorescent tumors with constant color-map limits from 1 to 16 ns. This facilitated a convenient visual inter-specimen interpretability of images. Also, brain parenchyma was visualized in blue and strong PpIX fluorescence in red, which is in accordance with what surgeons are used to from conventional PpIX-guided surgery. Intermediate lifetimes for PpIX at the order of magnitude of tissue autofluorescence could then be contrasted according to their magnitude by turquoise, green, yellow, and orange shades. Fluorescence intensity measurements were visualized as grayscale images with the color-map limits being scaled from 0 to the maximum intensity within the image.

We also blended the color-coded fluorescence lifetime images onto the respective fluorescence intensity images of the same region, which allowed for visualizing tissue structure and the fluorescence lifetime in a single image. In particular, our algorithm implemented in Python involved the following processing steps. Both input images were converted from RGB to HSL (hue, saturation, lightness) color space and hue and saturation from the intensity image were replaced with the respective values from the lifetime image. The resulting image was then back-converted to RGB and subsequently converted to YPbPr color space. The chrominances Pb and Pr were kept, and the luminance Y was replaced by the luminance of the original intensity image, which again was obtained by a RGB to YPbPr conversion.

## Results

### FD-FLIM Delineates Weak PpIX Fluorescence From Tissue Autofluorescence on Macroscale

**Figure 2:**
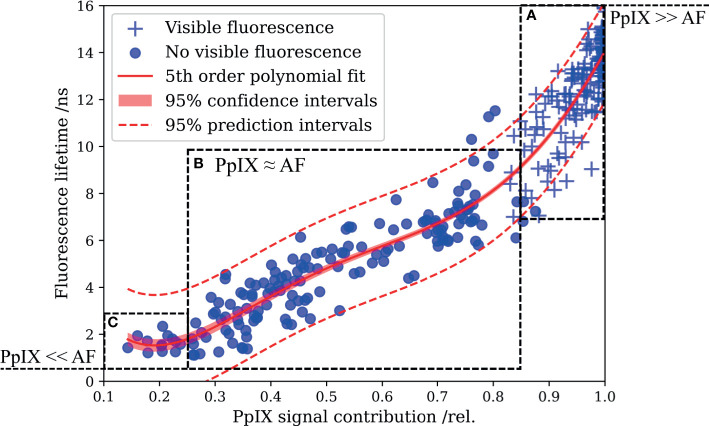
Correlating the fluorescence lifetime with the relative PpIX signal contribution (RSC_PpIX_) obtained from spatially registered spectroscopic measurements reveals the enhanced delineation of PpIX from tissue autofluorescence by means of FD-FLIM. **(A)** PpIX fluorescence exceeds tissue autofluorescence, and vague to strong intraoperative fluorescence was perceived from about 0.85 to 1.0 RSC_PpIX_. **(B)** Autofluorescence dominates the overall signal and tissue lacked intraoperative PpIX fluorescence. Increased fluorescence lifetimes facilitate the delineation of PpIX fluorescence from the overall autofluorescence background. **(C)** Fluorescence lifetimes in the magnitude expected for tissue autofluorescence were measured for small RSC_PpIX_ < 0.25.

In FD-FLIM, the measured lifetime is a composed average of the lifetimes of all excited and detected fluorophore emissions, weighted by their respective signal contributions. When measuring in 5-ALA-labeled tissue, both the autofluorescence and PpIX contribute to the overall fluorescence signal. To investigate the potential of delineating weak PpIX from the autofluorescence background, we combined FD-FLIM with spatially registered spectroscopic measurements. This allowed us to correlate the RSC_PpIX_ with the fluorescence lifetime, measured *ex vivo* in human brain tumor specimens. Three hundred thirty-one data points from 99 samples were evaluated, and a fifth-order polynomial fit together with CI and PI is shown in **Figure 2**. With the defined metric, strong intraoperative PpIX fluorescence was condensed at an RSC_PpIX_ of 1.0, with the fit indicating lifetimes of 14.1 ns ± 0.2 ns and 14.1 ns ± 2.1 ns for CI and PI, respectively. PpIX intraoperative fluorescence started becoming visible for RSC_PpIX_ of about 0.85 to 0.9, indicated through ROI A. This corresponded to lifetimes of about 8 to 16 ns, while the range from about 8 to 11ns mostly showed vague visible fluorescence. In ROI A, PpIX fluorescence was dominant respective to the autofluorescence background and could be visualized intraoperatively with a surgical microscope. More importantly, the interesting working range of FD-FLIM is highlighted in ROI B for RSC_PpIX_ from about 0.25 to 0.85. PpIX fluorescence was outweighed by autofluorescence and not visible during surgical resection. Yet, an increase in the RSC_PpIX_ led to an increase of the measured lifetime, facilitating the delineation of very weak PpIX fluorescence from the autofluorescence background. In ROI B, LGG and weakly infiltrated areas of other tumor entities as HGG and MET were found. Below an RSC_PpIX_ of 0.25 (ROI C), the fluorescence lifetime mostly stayed < 2 ns, which is on the order of magnitude expected for the autofluorescence of non-pathological brain parenchyma. Note that ROI C entailed very weak PpIX concentrations, which is examined in the context of methods for spectroscopic PpIX quantification in the discussion section.

### Selected Clinical Cases Illustrate the Value of FD-FLIM for Tumor Delineation

**Figure 3:**
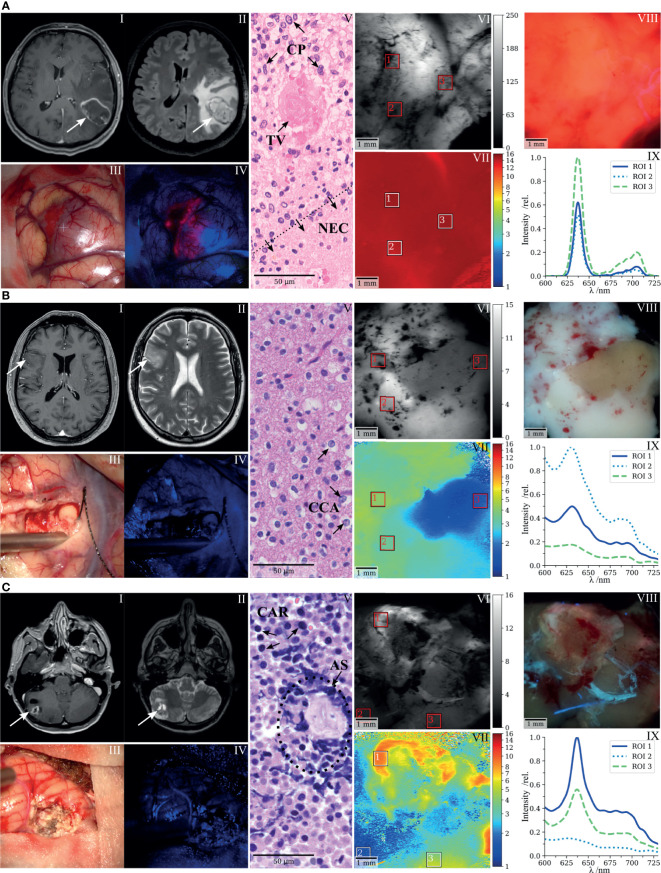
FD-FLIM delineation of PpIX and spectroscopic co-validation illustrated with three selected clinical cases. **(A)** Glioblastoma, **(B)** low-grade glioma, and **(C)** bronchial carcinoma metastasized to the cerebellum. (I,II) Preoperative T1- and T2-weighted MRI, (III,IV) surgical microscope white light and BLUE 400 fluorescence images taken during cytoreductive surgery, (V) representative histopathological section stained with hematoxylin and eosin, (VI,VII) demodulated fluorescence intensity (in mV_RMS_) and fluorescence lifetime (in ns) acquired on resected specimens with the raster-scanning FD-FLIM system, (VIII) fluorescence image of the respective specimens acquired with a scientific camera, and (IX) spectra acquired on the indicated ROIs in VI and VII; the fluorescence intensity (relative units) is plotted as a function of wavelength (in nm); CP, cellular pleomorphism; TV, thrombosed vessel; NEC, necrosis; CCA, clear-celled appearance; CAR, carcinoma cells; AS, artificially squeezed tissue.

In the previous section, we illustrated the full scope of FD-FLIM-enhanced tumor delineation by spectroscopic co-validation. Here, we provide a concise visualization of these findings with three selected clinical cases. **Figure 3A** shows an IDH-wild-type glioblastoma representing tumorous tissue commonly found within ROI A in **Figure 2**. Preoperatively MR images of the lesion in the left temporal lobe were performed demonstrating (I) a typical ring-like contrast enhancement in T1-weighted sequences and (II) a hyperintense appearance in FLAIR sequences. Intraoperative white-light and fluorescence surgical microscope images are shown in (III, IV), respectively. Strong intraoperative PpIX fluorescence was observed during surgery. Histopathology described characteristic cellular pleomorphism (CP), necrosis, and thrombosed vessels (TV; **Figure 3A**, V). Demodulated fluorescence intensity (in mV_RMS_) and fluorescence lifetime (in ns) maps are shown in (VI, VII). The average lifetimes from ROI 1 to 3 were 14.8, 13.9, and 14.8 ns, respectively. Strong visible PpIX fluorescence of the resected specimen was confirmed by a scientific camera (VIII) while rapidly scanning the laser across the tissue and setting the integration time to 2 s. Measured spectra for the respective ROIs are shown in (IX). The RSC_PpIX_ was 1.0 for each of the ROIs, with the PpIX fluorescence being orders of magnitude higher than tissue autofluorescence.

**Figure 3B** shows a representative case of an LGG. The preoperative MR images of the right fronto-temporal tumor showed (I) no significant contrast enhancement on T1-weighted sequences, but (II) appeared as a hyperintense lesion on T2-weighted sequences. No visible fluorescence was observed during surgery (IV). Note that the ZEISS BLUE 400™ emission filter is partially transmissive to the excitation light to highlight the tissue structure in blue. The representative hematoxylin and eosin (H&E) stain entailed central nervous system tissue with infiltrates of an IDH-mutant diffuse glioma with clear-celled appearance (CCA; **Figure 3B**, V). Fluorescence lifetimes (VII) acquired on a resected specimen were increased in ROI 1 and 2 (4.1 and 3.4 ns). ROI 3 (1.6 ns) was chosen on a region with lifetimes on the order of magnitude of tissue autofluorescence, corresponding to what we observed for samples within ROI C of **Figure 2**. Spectroscopic measurements (IX) confirmed the accumulation of PpIX in ROI 1 and 2, with an RSC_PpIX_ of 0.37 and 0.31, respectively. RSC_PpIX_ on ROI 3 was 0.24 with very weak absolute PpIX fluorescence. In the camera image (VIII), PpIX fluorescence was outweighed by autofluorescence and a brighter white and a less intense region could be observed while integrating for 2 s.

**Figure 3C** depicts a metastasis of a bronchial carcinoma located in the right cerebellum. Preoperative MR images of the tumor demonstrated (I) contrast enhancement in T1-weighted sequences and (II) a hyperintense signal in T2-weighted sequences. Intraoperatively, no fluorescence was visible (IV). The histopathological workup described small, partly necrotic fragments of a carcinoma (CAR) with small- to middle-sized cells and expression of neuroendocrine markers (**Figure 3C**, V). Tissue partly appeared artificially squeezed due to the surgical intervention (AS). When integrating for 10 s with the scientific camera, slight PpIX fluorescence became visible in the upper and right-hand part of the resected sample (VIII). The fluorescence lifetime on the corresponding ROI 1 was 8.5 ns (VII), with the RSC_PpIX_ being 0.68 (VIII). PpIX fluorescence within ROI 1 therefore was slightly below the threshold for visible intraoperative fluorescence, considering the findings from **Figure 2**. ROI 3 entailed a fluorescence lifetime and RSC_PpIX_ of 4.5 ns and 0.61, respectively. Here, autofluorescence was dominant but spectrally and temporally resolved fluorescence clearly delineated PpIX in the tissue. ROI 3 (1.5 ns, RSC_PpIX_ = 0.19) visualizes a region with very little PpIX accumulation and lifetimes on the order of magnitude of tissue autofluorescence.

### PpIX Fluorescence Lifetime Statistics of Gliomas, Meningiomas, and Metastasis

**Figure 4:**
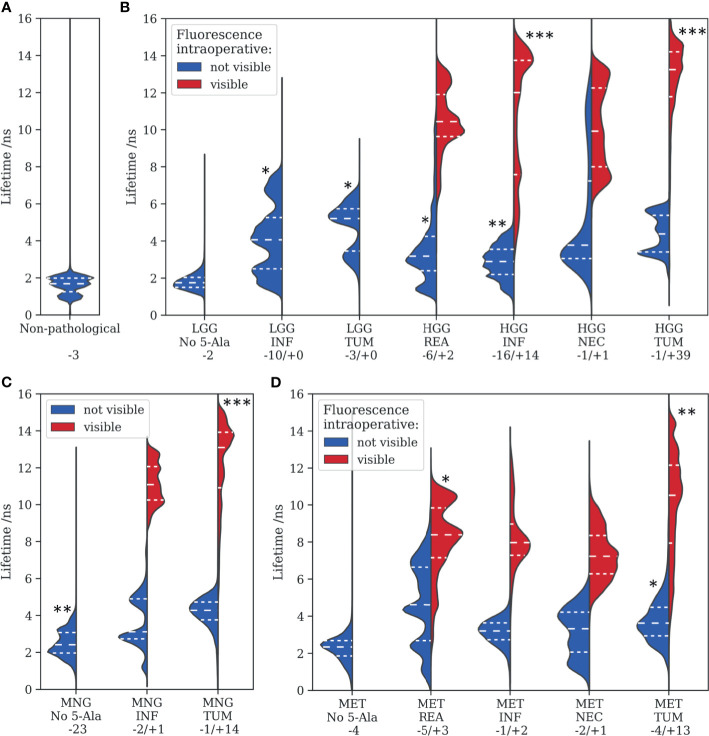
Fluorescence lifetimes measured for various tumor entities are visualized with violin plots, where the dashed lines represent the median, 0.25 and 0.75 quartiles. **(A)** non-pathological tissue, **(B)** low- and high-grade gliomas, **(C)** meningiomas, and **(D)** metastasis. Tissue was further categorized into reactive (REA), infiltration zones (INF), necrotic regions (NEC), and core tumor areas (TUM) according to the histopathological workup. Statistical levels of significant difference to **(A)** of each subgroup are indicated with asterisks (*<0.05, **<0.01, ***<0.005). Numbers after a “– or “+” refer to the amount of samples without or with visible intraoperative fluorescence, respectively.

This section provides an overview of the fluorescence lifetimes measured for all specimens, including non-pathological tissue, LGG, HGG, MNG, and MET (**Figures 4A–D**). Tissue was further classified as reactive, infiltrated, necrotic, or core tumor, according to the histopathological workup. The descriptive statistics are visualized in violin plots, with the median and first and third quartiles indicated through dashed lines. Groups were tested for statistical significant differences against the non-pathological group, with the alternate hypothesis being that the median fluorescence lifetime was greater than what we observed in (A). Most importantly, both infiltration zones (median = 4.1 ns, first quantile = 2.5 ns, third quantile = 5.3 ns, *p = 0.017*) and core tumor areas (5.2 ns, 3.5 ns, 5.7 ns, *p = 0.040*) of LGG showed significantly increased lifetimes respective to the control group (1.7 ns, 1.3 ns, 2.0 ns). None of the LGG showed visible fluorescence during surgery. In infiltrated parenchyma of HGG, the number of samples for visible and non-visible intraoperative fluorescence was balanced with significantly increased median lifetimes, 2.9 ns (*p = 0.008*) and 12.0 ns (*p = 0.005*), respectively. Note that infiltrated tissue often showed heterogeneous visible fluorescence. This explains why about 30% of the measured tumor area in the “visible” group was below the identified threshold (**Figure 2**) for visible fluorescence of about 8 ns. While the majority of reactive parenchyma in HGG was visually non-fluorescent, 39 of 40 core tumor specimens showed strong fluorescence and clearly increased lifetimes (13.2 ns, 11.8 ns, 14.2 ns, *p = 0.002*). It is interesting to note that many MNG tumor specimens exhibited strong fluorescence with increased lifetimes (13.1 ns, 10.9 ns, 13.9 ns, *p = 0.005*), although a vast majority of those tumors was classified as benign (WHO grade I, **Supplementary Table 6**). Likewise, increased lifetimes were found for different tissue types of brain metastasis. Autofluorescence lifetimes of tumorous tissue without 5-ALA were clearly increased for both MNG (2.4 ns, 2.0 ns, 3.1 ns, *p = 0.010*) and MET (2.3 ns, 1.9 ns, 2.7 ns, *p = 0.056*), respective to autofluorescence lifetimes of physiological brain. Please refer to **Supplementary Table 2** for descriptive and inferential statistics of all tumor entities.

### FD-FLIM and Structural Tissue Visualization in Neurosurgical Microscopes

**Figure 5:**
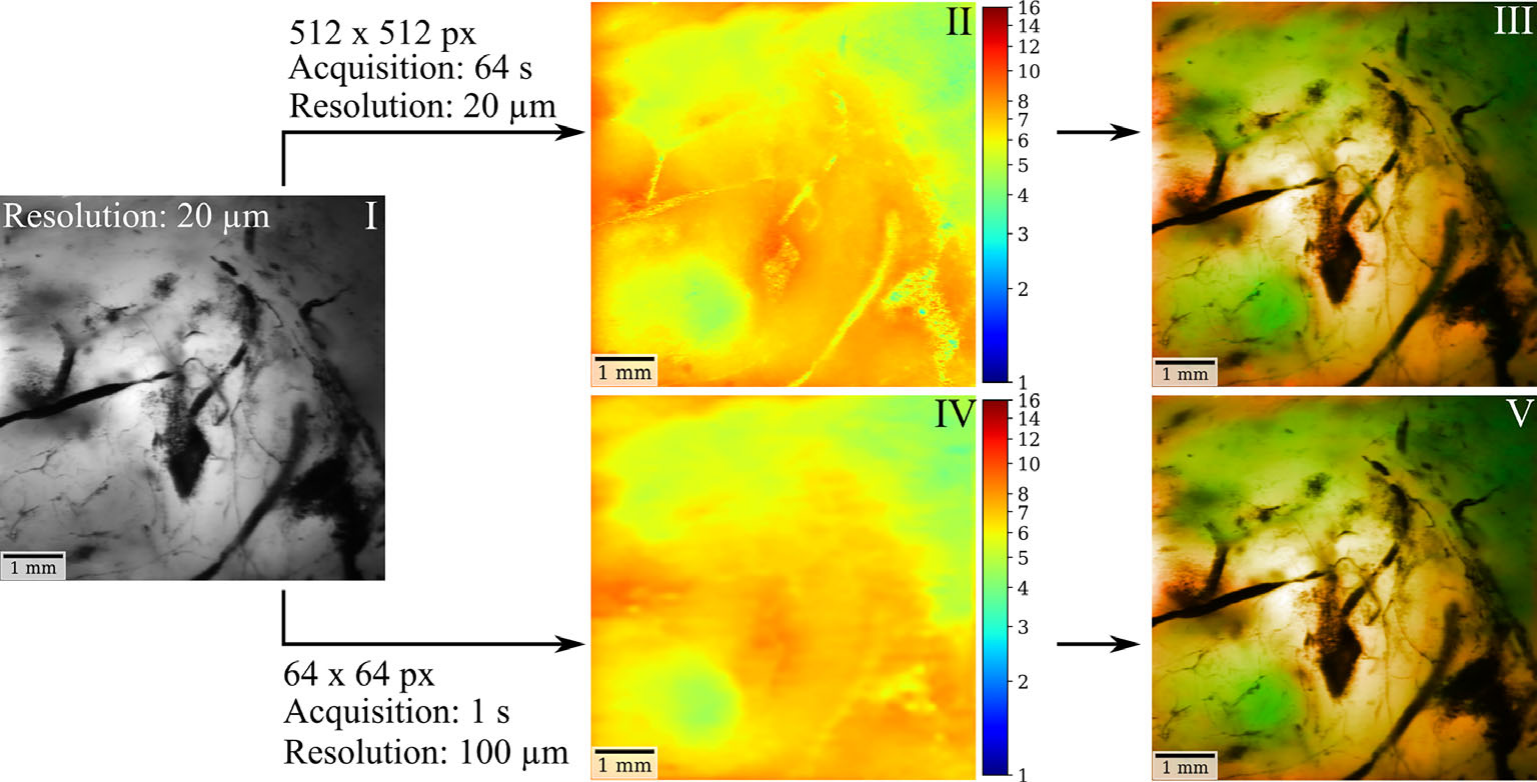
FD-FLIM and structural tissue visualization in surgical microscopes can be achieved through color blending in near real time. Using a highly resolved fluorescence intensity (I) or a white-light camera image as a basis, color-blending the fluorescence lifetime (II) provides both structural tissue and lifetime information (III). When reducing the resolution of the FD-FLIM image to 100 µm (IV) acquisition time could be decreased to 1 sec. The corresponding color-blended image (V) yields comparable contrast to (III), with the perceived image quality and resolution being dominated by the luminance of the intensity image.

In the previous sections, we outlined the capacity of FD-FLIM to delineate LGG and weakly fluorescent tumorous tissue on macroscale. The second imperative for successful clinical translation is the coherence with surgical instrumentation and workflows. This section therefore covers important aspects related to engineering the technology toward clinical translation. For the simultaneous visualization of both tissue structure and fluorescence lifetime, we suggest color-blending FD-FLIM measurements on either a fluorescence intensity or a white-light camera image. The overlay algorithm is described in more detail in *Materials and Methods*. **Figure 5** illustrates how color-blending a fluorescence lifetime (II) on a highly resolved fluorescence intensity image (I) provides both tissue morphology and FD-FLIM contrast (III). In parallel, fusing lifetime and structural information is highly beneficial in terms of measurement speed. While the homodyne detection of FD-FLIM allows to recover weak signals from noise, the photon budget in surgical microscopes with long working distances is limited. This entails a trade-off between the field of view (FOV), the lateral resolution, and the frame rate. When reducing the resolution of the FD-FLIM image to 100 µm (IV, 64 × 64 pixels), acquisition time decreased to 1 s. The respective color-blended image (V) was highly comparable to (III), and the perceived image resolution was dominated by the luminance of the structural tissue image. For quantifying the similarity between II and IV as well as III and V, we employed the structural similarity index measure (SSIM). The SSIM takes both the perceived alteration of structural information as well as changes in luminance and contrast into account. While the SSIM was 0.895 for II and IV, it was 0.986 for III and V, confirming the visually perceived similarity. Note that when comparing two identical images, the SSIM would be 1.0.

For information on the measurement accuracy of FD-FLIM in tissue and the extent of PpIX bleaching under prolonged excitation, please refer to **Supplementary Figures 2** and **3**. We also propose an adjusted implementation of FD-FLIM for surgical microscopes with variable working distances (**Supplementary Materials Section 1.1**).

## Discussion

Maximal safe resection is of decisive importance in the management of brain tumors. PpIX fluorescence-guided surgery has improved neurosurgical resection in HGG but lacks sensitivity and specificity for the delineation of LGG and weakly infiltrated tumor areas of HGG. Our results emphasize the capacity of FD-FLIM to detect weak PpIX fluorescence and distinguish it from spectrally overlapping tissue autofluorescence on macroscale.

The benchmark for sensitive PpIX detection in tissue has been set by spectroscopic PpIX quantification, implemented both in handheld probes (7, 21) and in hyperspectral wide-field setups integrated in surgical microscopes (36, 40). While measuring spectrally resolved fluorescence facilitates clear distinction between weak PpIX and tissue autofluorescence, the technique has not been translated to clinical practice yet. This might be contributed to the size of the instrumentation and the spectral complexity of different physiochemical states of PpIX which hamper PpIX quantification especially in LGG (41). Wide-field PpIX quantification is also challenged by inhomogeneous illumination profiles across the field of view or resection cavities being not flat (42). The fluorescence lifetime is to a good degree parametrically independent to such perturbations as it is an intrinsic property of fluorophores (26). In this work, we combined fluorescence lifetime measurements of PpIX in brain tumor specimens with spatially registered spectroscopic measurements for the first time. We correlated FD-FLIM measurements with the respective PpIX signal contribution as a ground truth. Autofluorescence lifetimes were found to be in the range of about 0.8 to 2 ns (32, 33), which was substantiated by a non-pathological sample within this study. The multimodal approach revealed how even weak PpIX concentrations in tissue led to an increase of the measured lifetime respective to tissue autofluorescence. While most HGG core tumor specimens exhibited strong intraoperative fluorescence, autofluorescence became dominant for LGG and weakly infiltrated, reactive, or necrotic tissue of HGG, MNG, and MET. At about 0.85 RSC_PpIX_ and below, PpIX was outweighed by the autofluorescence background and not visible during surgical resection. Down to an RSC_PpIX_ of about 0.25, the multimodal approach demonstrated enhanced FD-FLIM tumor delineation. The variations observed within the prediction intervals in **Figure 2** were expected as the autofluorescence background intensity and lifetimes varied among samples. This can be contributed to differences in absorption and scattering as well as to the known heterogeneity of metabolic strategies employed by brain tumors (43). Furthermore, porphyrins such as uroporphyrin and coproporphyrin (44) and various photoproducts of PpIX with shorter lifetimes (45) likely contributed to the overall signal.

Studies quantifying PpIX concentrations in tissue account for optical tissue properties as absorption and scattering and employ spectral unmixing to isolate the PpIX signal from the background fluorescence or photoproducts of PpIX (46). The relation between the PpIX concentration and the RSC_PpIX_ of the raw signal is therefore inherently non-linear. Nevertheless, we consider it helpful to put our work into this context, as the comparison enables the reader to get an estimate of the sensitivity of time-resolved fluorescence measurements in respect to spectroscopic PpIX quantification. It is worth mentioning that an RSC_PpIX_ below 0.25 was most likely linked to extremely weak PpIX concentrations. Widhalm et al. presented spectra for two LGG with PpIX being quantified to 13 and 35 ng/ml (7). We evaluated the RSC_PpIX_ in the corresponding processed spectra to be about 0.37 and 0.55, respectively. The non-pathological cortex entailed a PpIX concentration of 1 ng/ml, with a small peak corresponding to about 0.09 RSC_PpIX_ (7). Similarly, Valdés et al. showed spectra of two LGG with concentrations of 56 ng/ml (21) and 82 ng/ml (47), corresponding to about 0.57 and 0.67 RSC_PpIX_, respectively. This suggests that the low concentrations within the presented LGG could have been contrasted by FD-FLIM. Note that reactive tissue accumulates PpIX and entails increased lifetimes which might be mistaken for infiltrated tumor regions. This might be seen as a general limitation for the specificity of methods basing their contrast on PpIX fluorescence. Combining FD-FLIM with other imaging modalities could provide valuable complementary information. A possible workflow might involve wide-field screening of the resection cavity with FD-FLIM and subsequent co-validation in the operating room by methods such as stimulated Raman histology (48) or confocal laser endomicroscopy (49, 50).

The proposed optical fiber-based architecture facilitates the integration of FD-FLIM into surgical microscopes with minor space requirements, which outlines the coherence of our method with surgical workflows. Nevertheless, the low collection efficiency at long working distances remains challenging for real-time visualization. Our setup allows for acquiring highly resolved (25 µm lateral resolution, 6.5 × 6.5 mm^2^ FOV) FD-FLIM images at a working distance of 200 mm in about 16 s. This would allow for screening the resection cavity for residual tumor with a reasonable acquisition time. For enhanced visualization, we proposed to fuse FD-FLIM maps with a reduced lateral resolution with highly resolved white-light or fluorescence intensity images. With the proposed algorithm, tissue structure is visualized with high resolution and fluorescence lifetime contrast is provided with sufficient accuracy. In our setup, a reduction of the lateral FD-FLIM resolution to about 100 µm reduced the acquisition time to 1 s. A further reduction of the FD-FLIM resolution would be possible without sacrificing relevant information. Note that other approaches such as hand-held scanning with a point measurement probe achieved accuracies of 1 to 2 mm, with the need to correct for tissue motion with sophisticated algorithms (51). In the same manner, FD-FLIM scans could be acquired for the entire FOV within fractions of a second and then overlaid onto a continuous white-light video stream. The combination of image processing together with surgical microscopes designed with an optimized detection efficiency in the FLIM channel is promising for real-time intrasurgical FD-FLIM.

Apart from the capacity for sensitive intrasurgical tumor delineation, we found the value of PpIX lifetime imaging to be in the ease of interpretation. Independently of factors as excitation intensity and the FD-FLIM measurement instrumentation, the measured lifetime in tissue can expected to be in the range of about 0.5 to 16.4 ns. While a simple threshold at around 2 ns would provide excellent separation between PpIX and tissue autofluorescence, FD-FLIM is also promising to provide a robust input to machine learning classification models. In future, data acquired with various systems across a multitude of clinical centers could be fused to provide a big database for enhanced tumor delineation and classification. Aside from PpIX, label-free approaches measuring the fluorescence lifetime of endogenous tissue fluorophores as FAD or NADH provide a potential source of contrast for intrasurgical brain tumor delineation. Studies employing time-resolved two-photon microscopy showed increased mean NADH lifetimes in human glioma cell lines compared to non-pathological brain (52, 53). Alfonso-Garcia et al. intraoperatively demonstrated increased lifetimes for necrotic tissue respective to cortex as well as decreased lifetimes for an oligodendroglioma patient when comparing to the lifetimes of adjacent white matter (54). Higher mean NADH fluorescence lifetimes were also observed in glioblastomas respective to physiological brain (27, 55). The interested reader is also referred to Marcu and Hartl (25). Further studies are required to better understand the potential of endogenous fluorescence lifetimes for tumor delineation in the brain, and a special focus should be set on LGG and infiltrated tissue at tumor boundaries. In this respect, the heterogeneity of metabolic strategies employed by gliomas is particularly challenging (43).

Alongside these promising aspects, this study has to be seen under the following limitations. First, measurements were performed *ex vivo*. Larger studies with intrasurgical measurements ultimately are required to investigate tissue *in vivo*. In this study, we intended to keep the state of tissue as close to *in vivo* as possible. We imaged within 1 h after resection and kept the specimens in artificial cerebrospinal fluid. More importantly, *ex vivo* imaging allowed us to validate FD-FLIM with spatially registered spectroscopy, as the long acquisition time of spectra and tissue motion would have complicated the method *in vivo*. Second, this implied a low number of non-pathological specimens, since biopsies few centimeters away from the tumor boundary are only feasible in very rare cases (for example the approach to deep-seated tumors). The small size of the control group in our study limited the explanatory power of statistical inference. Extended clinical trials will be necessary to account for this limitation. However, tissue in adequate distance to the MRI contrast-enhancing tumor boundary could be assumed to be non-pathological in future *in vivo* studies. While no histological confirmation would be possible without biopsy collection, we believe this approach to be reasonable for obtaining control measurements from the normal cortex. We emphasize that the value of this study lies in the multimodal co-validation, which coherently substantiates the capacity of FD-FLIM to delineate weak PpIX from the autofluorescence background. Third, the current laser power of 6 mW is compliant with laser safety norms (ANSI Z136.1) for tissue and safe for diffuse reflections toward the eye. However, specular reflections toward the eye could pose a problem in a worst-case scenario. As of now, at least optical density 1 laser safety protection is required. Considering a limiting aperture of 7 mm as specified for point-source ocular exposure, a maximum laser power of 1 mW is permissible at the eye. This suggests that the technology can be engineered toward an operation without any safety protection. Optimizing the surgical microscopes detection efficiency in the FLIM channel together with the proposed image processing methods would allow for decreasing the laser power. Alternatively, algorithmic safety measures can allow for detecting surgical instruments and reflecting surfaces based on the white-light video-stream and automatically shut down the laser before specular reflections can occur. Fourth, the homodyne detection inherent to FD-FLIM is to a good extent unaffected by unmodulated signal contributions as ambient light. Nevertheless, the relatively bright laboratory room light did increase the background signal at 10 MHz, which increased the noise and phase jitter for measurements of very weak tissue autofluorescence. We therefore turned off the light for an optimal signal-to-noise ratio. In the dimmed light conditions common to fluorescence-guided surgery, we would not expect this to pose a problem. Simultaneous white-light illumination during FD-FLIM measurements can be achieved by shaping the light source emission spectrum to not emit within the PpIX detection band.

To conclude, this study emphasizes the potential of FLIM to optimize maximal safe resection in brain tumors in the future and highlights its potential toward clinical translation. The advantage of PpIX for fluorescence guidance for tumor resection has been outlined also in other medical disciplines such as urology and gynecology. Evidently, the findings on FLIM for maximal safe tumor resection could initiate intensive research activities to be transferred to those areas as well.

## Data Availability Statement

The raw data supporting the conclusions of this article will be made available by the authors, without undue reservation.

## Ethics Statement

The studies involving human participants were reviewed and approved by the Ethics Committee of the Medical University of Vienna (EK419/2008 – Amendment 04/2018). The patients/participants provided their written informed consent to participate in this study.

## Author Contributions

GW, BK, and LW resected the tumor specimens. JG, TR, and AW performed the histopathological workup. BK, LW, and AL organized the biopsy handling and preparation. MW and CH provided the technical consultation for modifications on the surgical microscope. ME and DR set up the FD-FLIM system under supervision of AU and MA. DR implemented the spectroscopy channel, performed the measurements, analyzed the data, and wrote the manuscript. ME, WD, GW, and RL initiated the project. All authors contributed to the article and approved the submitted version.

## Funding

This project has received funding from the Austrian Christian Doppler Research Association as well as from the innovation board of the Carl Zeiss Meditec AG. TR is a recipient of a DOC Fellowship of the Austrian Academy of Sciences at the Division of Neuropathology and Neurochemistry (25262). JG is supported by an OeNB grant 16725 to AW. The financial support by the Austrian Federal Ministry for Digital and Economic Affairs and the National Foundation for Research, Technology and Development is gratefully acknowledged. This project has furthermore received funding from the European Union Horizon 2020 research and innovation program under the Marie Sklodowska-Curie grant agreement (MSCA grant 721766).

## Conflict of Interest

MW and CH are employees of Carl Zeiss Meditec AG, Oberkochen, Germany. GW received restricted travel grants from NX Development Corp.

The remaining authors declare that the research was conducted in the absence of any commercial or financial relationships that could be construed as a potential conflict of interest.

## Publisher’s Note

All claims expressed in this article are solely those of the authors and do not necessarily represent those of their affiliated organizations, or those of the publisher, the editors and the reviewers. Any product that may be evaluated in this article, or claim that may be made by its manufacturer, is not guaranteed or endorsed by the publisher.
