# Supplementary material for: Fluorescence Lifetime Imaging and Spectroscopic Co-Validation for Protoporphyrin IX-Guided Tumor Visualization in Neurosurgery
Source: Front Oncol. 2021 Sep 14;11:741303. doi: 10.3389/fonc.2021.741303 (PMC8476921; doi:10.3389/fonc.2021.741303)
Supplement: Supplementary file 1 [file DataSheet_1.pdf]

Supplementary Material

1 Supplementary Information

1.1 Implementation of FD-FLIM in surgical microscopes with variable working distances

In FD-FLIM measurements, the demodulated phase delay is composed of the fluorophore's lifetime, delays in the electronic signal cascade and the time of flight delay, which essentially is the delay induced through the time it takes light to travel from the excitation source to the fluorophore and from there to the detector. For a fixed working distance, electronic and time of flight delays can be accounted for by referencing. Neurosurgical microscopes, however, are designed to facilitate working distances from about 200 to 600 mm. Considering that the light travels back and forth, the optical path may vary by about 800 mm, a distance traveled by light in 2.7 ns. Time of flight induced phase delays therefore must be considered. We assume that electronic delays and the time of flight delay at an arbitrary zero plane have been corrected for by referencing. Whenever the working distance is changed, the corresponding change of optical path length can be extracted from the surgical microscope system. Using the speed of light, the respective time of flight is calculated for the additional optical path. As the relation between phase and fluorescence lifetime is nonlinear, the time of flight induced phase delay has to be calculated first. For the above example with an additional time of flight of 2.7 ns, the corresponding phase delay $\Phi_{ToF\,\Delta}$ would be about 9.5°. This can be calculated by

$$\Phi_{ToF\,\Delta} = \arctan\left(\tau_{ToF} \cdot 2\pi \cdot f_{Modulation}\right),$$

where $\tau_{ToF\,\Delta}$ is the additional time of flight respective to the zero reference plane and $f_{Modulation}$ the modulation frequency of the excitation light source. The corrected fluorescence lifetime τ can then be calculated by

$$\tau = \frac{\tan\left(\Phi_{demodulated} - \Phi_{ToF\,\Delta}\right)}{2\pi \cdot f_{Modulation}},$$

where $\Phi_{demodulated}$ is the measured phase delay by the FD-FLIM system.

Note that when imaging tissue with a topography deviating from the plane of best focus, these deviations also contribute to the overall time of flight. In practice, small deviations of several millimeters, however, can be neglected as the corresponding additional time of flight is on the order of 10 picoseconds.

2 Supplementary Figures and Tables

2.1 Supplementary Figures

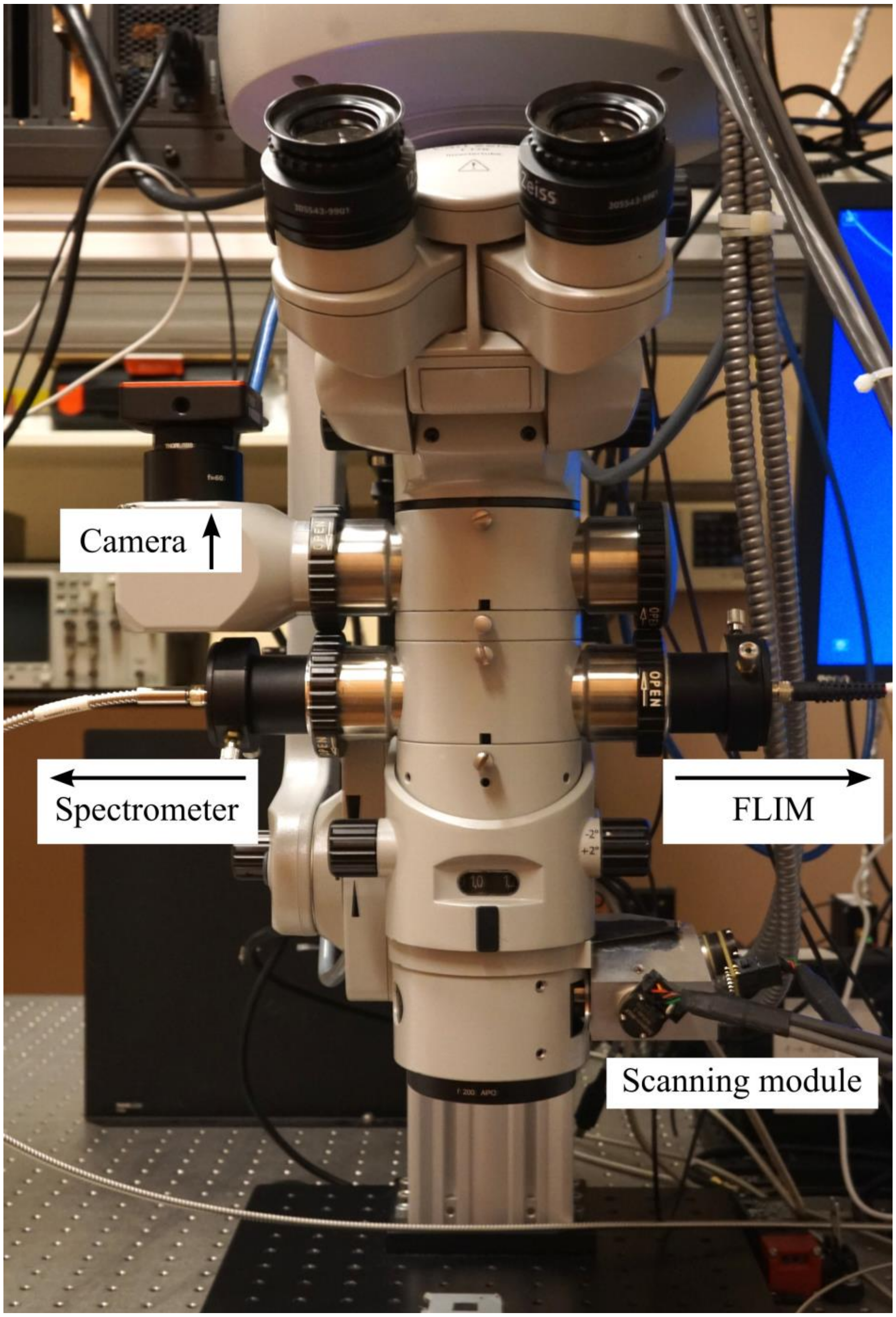

Supplementary Figure 1. Surgical microscope with integrated fluorescence lifetime imaging (FLIM) and spectrometer. A fiber from a 405 nm diode laser is attached to the scanning module (backside, not depicted). Fluorescence emission is collected by the main objective lens and guided through the stereoscopic paths into two fiber-coupled channels (FLIM, right; spectrometer, left). A camera can additionally acquire white-light and fluorescence images of the specimens.

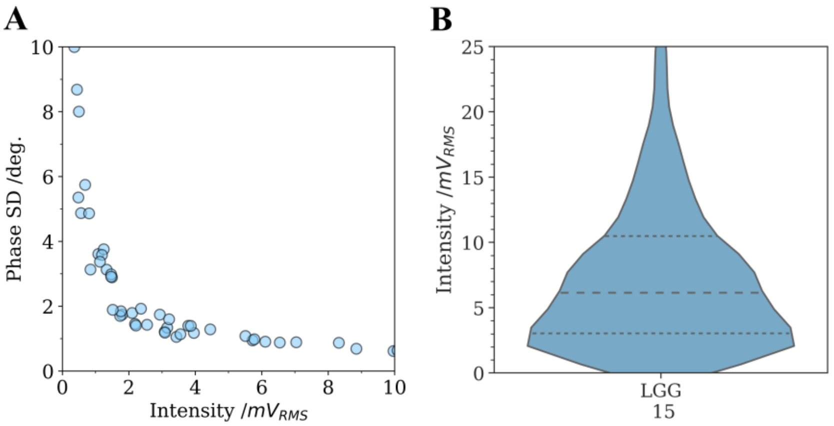

Supplementary Figure 2. (A) Phase standard deviation measured in tissue as a function of the demodulated fluorescence signal intensity from 0 to 10 mV_{RMS}. (B) Fluorescence intensity distribution in low-grade gliomas, with the median, 0.25 and 0.75 quartiles being indicated through dashed lines.

Accuracy of FD-FLIM measurements in tissue

When designing surgical microscopes for FD-FLIM integration, it is imperative to know how the numerical aperture (NA) for photon collection and laser power for fluorescence excitation impact the accuracy of FD-FLIM measurements in tissue. The below considerations were made for detection with a single stereo-path (NA = 0.04) and 6.0 mW power at the laser spot and can be scaled to other system configurations. In FD-FLIM, the measured phase entails stochastical variations which can be described by an average value and a corresponding standard deviation (SD). The magnitude of these variations on one hand can be decreased by increasing the lock-in amplifiers integration time, which is set to allow for sufficient settling within the pixel dwell time. This parameter therefore is a constant for a given setup, chosen in a trade-off with the required measurement speed. On the other hand, the phase SD in FD-FLIM measurements strongly depended on the intensity of the fluorescence signal. Fig. S2 A depicts the phase standard deviation measured in tissue as a function of the demodulated intensity from 0 to 10 mV_{RMS}. This is the range we observed in our setup for tissue autofluorescence or weak PpIX accumulations as found in LGG. Fig. S2 B shows the intensity distribution in LGG (median = 6.2 mV_{RMS}, Q1 = 3.0 mV_{RMS}, Q2 = 10.5 mV_{RMS}). Combining the information from (A, B), it is reasonable to assume a phase SD of about 1° for weakly fluorescent tissue with the above system configuration. Note that the phase is averaged in the course of a pixel dwell time and the phase error in practice will be somewhat smaller. Due to the nonlinear relation between phase and lifetime, the corresponding lifetime SD does depend on the actual value of the lifetime. At a fluorescence lifetime of 2.0 ns, a phase SD of 1° would result in a deviation of about 0.28 ns. This error averages out when measuring over multiple neighboring pixels. Low fluorescence signal intensities, however, will increase the noise in the lifetime measurements.

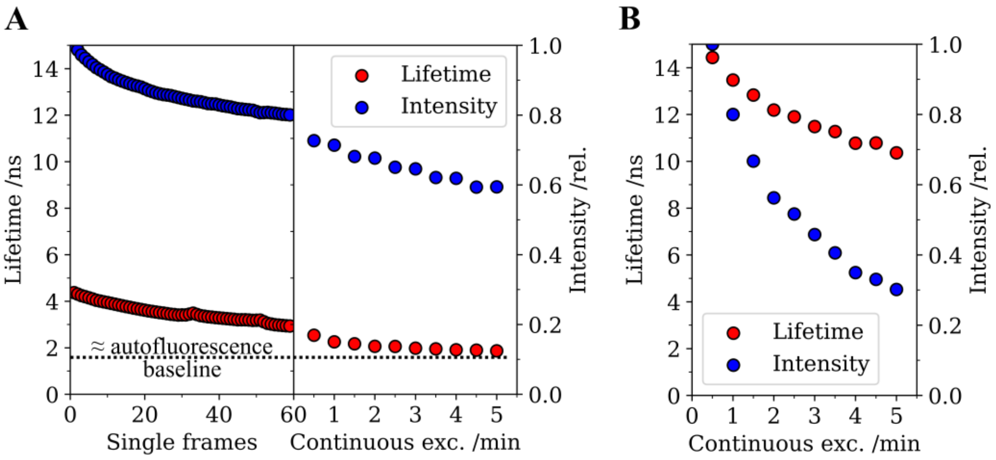

Supplementary Figure 3. Decrease of the fluorescence intensity and fluorescence lifetime due to bleaching of PpIX after the acquisition of 60 single frames and/or subsequent 5 minutes of continuous excitation (A) for a sample showing no visible PpIX fluorescence during surgery and (B) for a sample showing strong visual PpIX fluorescence.

Bleaching of PpIX fluorescence during FD-FLIM measurements

Fluorophore bleaching is a common issue in fluorescence intensity measurements. While the fluorescence lifetime is intrinsically independent of the fluorophore concentration, PpIX bleaches faster than tissue autofluorescence which in turn reduces the relative PpIX signal contribution (RSC_{PpIX}) and therefore the measured lifetime with increasing imaging time. To quantify the impact of bleaching on FD-FLIM measurements in tissue, we repeatedly imaged a tumor specimen which didn't show visible fluorescence during surgery and entailed an initial lifetime of about 4.4 ns (Fig. S3 A). We first acquired 60 single frames and then switched to 5 min of continuous excitation, acquiring fluorescence lifetime and intensity data every 30 seconds. The fluorescence intensity was bleached to 80 % of its initial value after the first 60 frames, and then further reduced to 59 % after 5 min of continuous excitation. It is reasonable to assume an initial RSC_{PpIX} of 35 to 40 %, which was completely bleached within the experiment, leaving the non-bleached tissue autofluorescence. This is supported by fluorescence lifetime measurements, where the initial lifetime of 4.4 ns reduced to 2.9 ns after the first 60 frames. After 5 min of continuous excitation, the fluorescence lifetime was further reduced to 1.9 ns, which is in the range expected for tissue autofluorescence. Such long periods of continuous illumination are rare in practice, suggesting that there is sufficient time for the detection and resection of malignant tissue areas before bleaching of PpIX. In any case, a mixture of continuous FD-FLIM imaging and single frame screening of the resection cavity seems to be to the most practical workflow for weakly fluorescent tissue.

We also performed bleaching analysis on a strongly fluorescent HGG sample (Fig. S3 B). The initial average lifetime of 14.4 ns was reduced to 10.4 ns after 5 min of continuous excitation. Fluorescence intensity was bleached to 30 % of its initial value, indicating that PpIX fluorescence was orders of magnitude stronger than autofluorescence and remained the dominant signal contributor even after bleaching 70 % of the initial intensity.

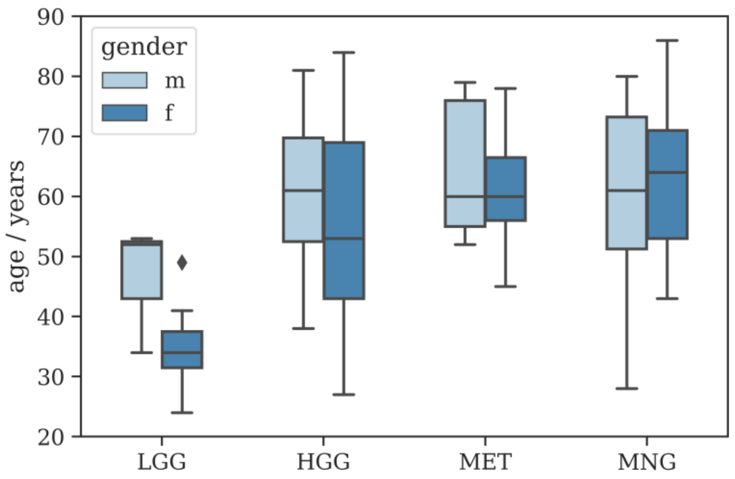

Supplementary Figure 4. Patient age distribution (in years) for female (f) and male (m) subjects included in the study. Patients are grouped by their respective tumor diagnosis (LGG: low-grade gliomas, HGG: high-grade gliomas, MET: metastasis, MNG: meningiomas)

2.2 Supplementary Tables

Supplementary Table 1. Median, 0.25 and 0.75 quartiles of the patient age distribution in years (f=female, m=male) corresponding to Supplementary Figure 4.

	**LGG**		**HGG**		**MET**		**MNG**	
**gender**	f	m	f	m	f	m	f	m
**N patients**	7	3	25	30	15	13	25	16
**median age**	34	52	53	61	60	60	64	61
**Q1**	32	43	43	53	56	55	53	51
**Q2**	38	53	69	70	67	76	71	73

Supplementary Table 2. Median, 0.25 and 0.75 quartiles (Q1, Q2), mean and standard deviation (SD) for fluorescence lifetimes (in ns) measured on non-pathological tissue (CTL), low-grade gliomas (LGG), high-grade gliomas (HGG), meningiomas (MNG) and metastasis (MET) specimens. No 5-ALA: no administration of 5-ALA prior to surgery, INF: infiltrated parenchyma, TUM: core tumor, REA: reactive tissue, NEC: necrotic areas, '-': no visible fluorescence during surgery, '+': visible fluorescence during surgery; p-values were calculated for each subgroup respective to the control group by employing a non-parametric Mann-Whitney-U test. To assure equal contribution of all specimen, the number of evaluated pixels per sample was normalized to the smallest specimen. This resulted in 21 733 randomly selected pixels per specimen being included in the analysis.

	**CTL**	**LGG**			**HGG**							
**subgroup**		No 5-ALA	INF-	TUM-	REA-	REA+	INF-	INF+	NEC-	NEC+	TUM-	TUM+
**N samples**	3	2	10	3	6	2	16	14	1	1	1	39
**p-value**	-	0.614	0.017	0.040	0.014	0.074	0.008	0.005	0.186	0.186	0.186	0.002
**median** τ	1.7	1.7	4.1	5.2	3.2	10.4	2.9	12.0	3.8	9.9	4.4	13.2
**Q1**	1.3	1.5	2.5	3.5	2.4	9.6	2.2	7.6	3.1	8.0	3.4	11.8
**Q2**	2.0	2.0	5.3	5.7	4.3	11.9	3.6	13.8	7.3	12.3	5.4	14.2
**mean**	1.6	1.8	4.1	4.8	3.7	4.4	2.9	10.7	5.3	10.1	4.4	12.6
**SD**	0.5	0.4	1.8	1.3	2.1	1.0	0.9	3.5	3.2	2.2	1.0	2.4

	**MNG**					**MET**								
**subgroup**	No 5-ALA	INF-	INF+	TUM-	TUM+	No 5-ALA	REA-	REA+	INF-	INF+	NEC-	NEC+	TUM-	TUM+
**N samples**	23	2	1	1	14	4	5	3	1	2	2	1	4	13
**p-value**	0.010	0.074	0.186	0.186	0.005	0.056	0.068	0.040	0.186	0.074	0.074	0.186	0.026	0.005
**median** τ	2.4	3.1	11.1	4.3	13.1	2.3	4.6	8.4	3.2	8.0	3.3	7.2	3.6	10.5
**Q1**	2.0	2.7	10.3	3.8	10.9	1.9	2.7	7.2	2.7	7.3	2.1	6.3	3.0	8.0
**Q2**	3.1	4.9	12.1	4.7	13.9	2.7	6.6	9.8	3.6	9.0	4.2	8.4	4.5	12.2
**mean**	2.5	3.7	11.2	4.2	12.2	2.3	4.7	8.2	3.2	8.3	3.2	7.3	3.8	10.0
**SD**	0.8	1.4	1.1	0.9	2.4	0.7	2.2	2.0	0.7	1.7	1.3	1.4	1.2	2.9

Tab. S3 to S6 provide an overview over all specimens for low-grade gliomas, high-grade gliomas, metastasis and meningiomas, respectively.

Supplementary Table 3. Overview over specimens resected from low-grade glioma surgery. #: Patient number; Hist.: histological tissue classification; Fluorescence status: '-': no visible fluorescence during surgery, '+': visible fluorescence during surgery, No 5-ALA: no administration of 5-ALA prior to surgery; Mean τ: average fluorescence lifetime, SD τ: standard deviation fluorescence lifetime

#	Age	Gender	Hist.	Diagnosis	Fluo. status	Mean τ /ns	SD τ /ns
1	53	m	INF	Oligodendroglioma WHO II	-	2.5	0.5
2.1	34	m	INF	Diffuse astrocytoma WHO II	-	2.4	0.4
2.2			TUM	Diffuse astrocytoma WHO II	-	5.8	0.5
2.3			TUM	Diffuse astrocytoma WHO II	-	5.5	0.5
3	41	f	INF	Astrocytoma WHO II	-	1.7	0.3
4	52	m	INF	Diffuse oligodendroglioma WHO II	-	3.0	0.9
5	30	f	TUM	Pilozytic astrocytoma WHO I	No 5-ALA	1.6	0.3
6.1	34	f	INF	Anaplastic astrocytoma WHO II	-	6.5	0.3
6.2			INF	Anaplastic astrocytoma WHO II	-	7.3	0.4
7.1	33	f	INF	Astrocytoma	-	5.2	0.3
7.2			INF	Astrocytoma	-	4.1	0.8
8	24	f	TUM	Diffuse astrocytoma WHO II	-	3.1	0.6
9.1	34	f	INF	Diffuse astrocytoma WHO II	-	4.5	0.3
9.2			INF	Diffuse astrocytoma WHO II	-	4.0	0.6
10	49	w	INF	Diffuse astrocytoma WHO II	No 5-ALA	2.0	0.5
**Total**	**N patients = 10, N specimens = 15**						

Supplementary Table 4. Overview over specimens resected from high-grade glioma surgery. #: Patient number; Hist.: histological tissue classification; Fluorescence status: '-': no visible fluorescence during surgery, '+': visible fluorescence during surgery, Mean τ: average fluorescence lifetime, SD τ: standard deviation fluorescence lifetime

#	Age	Gender	Hist.	Diagnosis	Fluo. status	Mean τ /ns	SD τ /ns
1	80	m	INF	Glioblastoma WHO IV	+	7.4	1.0
2	67	m	TUM	Glioblastoma WHO IV	+	14.5	0.7
3	75	m	INF	Glioblastoma WHO IV	+	5.5	0.9
4	31	f	INF	Glioblastoma WHO IV	-	2.4	0.7
5.1	49	m	REA	Glioblastoma WHO IV	-	4.0	1.3
5.2			TUM	Glioblastoma WHO IV	+	12.8	0.4
6	61	m	INF	Glioblastoma WHO IV	+	6.0	1.2
7.1	67	m	INF	Glioblastoma WHO IV	+	8.7	1.8
7.2			TUM	Glioblastoma WHO IV	+	13.4	0.5
8.1	62	m	INF	Glioblastoma WHO IV	-	1.9	0.4
8.2			TUM	Glioblastoma WHO IV	+	10.4	1.4
9.1	70	m	CTL	Non-pathological, resected on access route to glioblastoma	-	1.2	0.4
9.2			TUM	Glioblastoma WHO IV	+	12.3	1.4
10	59	m	TUM	Anaplastic astrocytoma WHO III	+	12.1	0.6
11.1	70	f	INF	Glioblastoma WHO IV	-	3.2	0.3
11.2			TUM	Glioblastoma WHO IV	+	12.2	2.1
12	59	f	TUM	Glioblastoma WHO IV	+	10.3	1.8

13	47	m	TUM	Glioblastoma WHO IV	+	10.0	2.3
14	66	m	TUM	Glioblastoma WHO IV	+	8.4	1.9
15.1	81	m	INF	Glioblastoma WHO IV	-	3.1	0.5
15.2			INF	Glioblastoma WHO IV	+	6.3	2.2
16	56	m	INF	Anaplastic astrocytoma WHO III	-	3.4	0.4
17.1	52	m	TUM	Glioblastoma WHO IV	+	12.8	0.6
17.2			TUM	Glioblastoma WHO IV	+	13.7	0.3
18	32	f	INF	Glioblastoma WHO IV	+	11.6	1.2
19.1	34	f	REA	Anaplastic astroblastoma	-	5.4	2.3
19.2			TUM	Anaplastic astroblastoma	+	13.8	0.3
20	51	f	TUM	Glioblastoma WHO IV	+	11.8	1.8
21	54	f	REA	Glioblastoma WHO IV	+	11.5	1.6
22	72	f	TUM	Glioblastoma WHO IV	+	11.4	2.3
23	84	f	TUM	Glioblastoma WHO IV	+	4.6	2.1
24	45	f	REA	Secondary glioblastoma	+	9.5	1.1
25.1	54	m	TUM	Glioblastoma WHO IV	-	4.4	1.0
25.2			TUM	Glioblastoma WHO IV	+	11.1	1.8
25.3			TUM	Glioblastoma WHO IV	+	14.5	0.3
26.1	61	m	TUM	Gliosarcoma WHO IV	+	10.9	2.0
26.2			TUM	Gliosarcoma WHO IV	+	11.8	0.7
27	50	f	REA	Gliosarcoma recurrent	-	5.4	2.2
28	71	f	TUM	Glioblastoma WHO IV	+	12.2	2.7

29.1	27	f	TUM	Secondary glioblastoma	+	12.5	0.5
29.2			INF	Secondary glioblastoma	-	3.4	0.7
30.1	67	f	TUM	Glioblastoma WHO IV	+	11.1	1.7
30.2			NEK	Glioblastoma WHO IV	-	5.3	3.2
31	69	f	REA	Glioblastoma WHO IV	-	2.0	0.6
32	75	m	REA	Glioblastoma WHO IV	-	2.8	0.8
33.1	57	m	TUM	Glioblastoma recurrent	+	11.9	0.6
33.2			REA	Glioblastoma recurrent	-	2.3	0.9
34	53	f	TUM	Glioblastoma recurrent	+	13.4	1.2
35.1	43	m	INF	Glioblastoma WHO IV	-	2.9	0.7
35.2			TUM	Glioblastoma WHO IV	+	14.5	0.3
35.3			INF	Glioblastoma WHO IV	+	13.5	0.5
36.1	73	m	TUM	Glioblastoma WHO IV	+	13.4	1.0
36.2			INF	Glioblastoma WHO IV	-	2.8	0.5
37.1	57	m	INF	Glioblastoma WHO IV	-	3.4	0.8
37.2			TUM	Glioblastoma WHO IV	+	13.9	0.8
38	44	m	INF	Glioblastoma recurrent	+	13.3	0.7
39	52	m	TUM	Glioblastoma recurrent	+	12.2	1.7
40	69	m	TUM	Glioblastoma WHO IV	+	13.6	1.2
41.1	43	f	INF	Anaplastic astrocytoma WHO III	-	1.7	0.6
41.2			INF	Anaplastic astrocytoma WHO III	-	4.2	0.4
41.3			INF	Anaplastic astrocytoma WHO III	-	2.6	0.7

41.4			INF	Anaplastic astrocytoma WHO III	-	2.6	0.9
42	49	f	INF	Glioblastoma WHO IV	+	9.7	2.0
43	38	f	TUM	Astrocytic glioma	+	14.1	0.3
44	60	f	TUM	Glioblastoma WHO IV	+	14.0	0.2
45	46	f	INF	Glioblastoma WHO IV	+	13.5	0.4
46.1	60	f	INF	Glioblastoma WHO IV	-	3.7	0.3
46.2			TUM	Malignant, glial tumor	+	14.5	0.3
47	66	m	INF	Glioblastoma WHO IV	+	12.6	2.3
48.1	39	f	INF	Glioblastoma WHO IV	+	14.5	0.3
48.2			INF	Glioblastoma WHO IV	-	2.4	1.5
49	74	f	INF	Glioblastoma WHO IV	+	13.3	1.1
50	38	m	INF	Glioblastoma WHO IV	+	13.8	1.2
51	77	f	TUM	Glioblastoma WHO IV	+	14.8	0.2
52	41	m	INF	Focal anaplastic astrozytoma	-	2.3	0.7
53	55	m	TUM	Glioblastoma WHO IV	+	15.5	0.6
54.1	74	m	TUM	Glioblastoma WHO IV	+	14.6	0.5
54.2			TUM	Glioblastoma WHO IV	+	15.0	0.4
55.1	72	m	NEC	Glioblastoma WHO IV	+	10.1	2.2
55.2			TUM	Glioblastoma WHO IV	+	14.0	0.9
55.3			TUM	Glioblastoma WHO IV	+	13.3	0.2
**Total**	**N patients = 55, N HGG specimens = 80, N control specimens = 1**						

Supplementary Table 5. Overview over specimens resected from brain metastasis surgery. #: Patient number; Hist.: histological tissue classification; Fluorescence status: '-': no visible fluorescence during surgery, '+': visible fluorescence during surgery, Mean τ: average fluorescence lifetime, SD τ: standard deviation fluorescence lifetime

#	Age	Gender	Hist.	Diagnosis	Fluo. status	Mean τ /ns	SD τ /ns
1.1	55	m	NEK	Metastasis lung cancer	-	2.2	1.0
1.2			REA	Metastasis adenocarcinoma	-	1.7	0.8
2.1	75	f	REA	Metastasis, primary seat lung or caecum	+	7.2	1.5
2.2			TUM	Metastasis adenocarcinoma	+	11.5	0.8
3	76	m	TUM	Metastasis adenocarcinoma	+	10.7	1.0
4	78	m	TUM	Metastasis adenocarcinoma	+	12.0	1.2
5	77	m	TUM	Metastasis Merkel cell carcinoma	-	3.5	1.0
6.1	52	m	REA	Metastasis adenocarcinoma	+	9.4	1.7
6.2			TUM	Metastasis adenocarcinoma	+	12.6	0.7
7.1	45	f	REA	Metastasis mamma carcinoma	-	6.0	0.7
7.2			TUM	Metastasis mamma carcinoma	+	7.1	1.4
7.3			TUM	Metastasis mamma carcinoma	+	7.4	1.4
8	60	f	TUM	Metastasis adenocarcinoma	-	3.5	0.7
9	60	m	TUM	Metastasis cardia carcinoma	+	10.0	1.2
10	51	f	REA	Metastasis adenocarcinoma	-	7.3	0.7
11	62	f	TUM	Metastasis bronchial carcinoma	-	3.8	1.8
12	77	f	TUM	Metastasis mamma carcinoma	No 5-ALA	2.2	0.7
13	67	m	TUM	Metastasis adenocarcinoma	+	9.1	2.0

14	58	f	TUM	Metastasis carcinoma	+	7.8	2.6
15	54	m	TUM	Metastasis lung cancer	+	14.3	0.4
16	78	f	TUM	Metastasis lung cancer	No 5-ALA	2.5	0.3
17	59	f	TUM	Metastasis thyroid cancer	No 5-ALA	2.4	0.8
18	70	f	NEK	Metastasis squamous cell carcinoma	-	4.2	0.7
19	60	f	NEK	Metastasis mamma carcinoma	+	7.3	1.4
20	55	m	TUM	Metastasis urachal carcinoma	+	12.5	1.1
21	58	m	TUM	Metastasis bronchial carcinoma	+	5.3	1.1
22	60	f	TUM	Metastasis bronchial carcinoma	No 5-ALA	1.9	0.8
23	63	f	INF	Metastasis renal cell carcinoma	+	8.9	2.2
24.1	56	m	TUM	Metastasis bronchial carcinoma	+	10.0	1.0
24.2			REA	Metastasis bronchial carcinoma	-	4.4	0.4
25.1	60	m	REA	Metastasis EBV-positive B-cell lymphoma	+	7.9	2.0
25.2			REA	Metastasis EBV-positive B-cell lymphoma	-	4.3	2.2
26.1	79	m	INF	Metastasis carcinoma	+	7.7	0.6
27	51		TUM	Metastasis tubulopapillary adenocarcinoma	-	4.4	1.0
28	54	f	INF	Metastasis mamma carcinoma	-	3.2	0.7
**Total**	**N patients = 28, N specimens = 35**						

Supplementary Table 6. Overview over specimens resected from brain meningioma surgery. #: Patient number; Hist.: histological tissue classification; Fluorescence status: ‘-‘: no visible fluorescence during surgery, ‘+’: visible fluorescence during surgery, Mean τ: average fluorescence lifetime, SD τ: standard deviation fluorescence lifetime

#	Age	Gender	Hist.	Diagnosis	Fluo. status	Mean τ /ns	SD τ /ns
1	74	m	TUM	Meningioma	No 5-ALA	1.4	0.4
2	43	f	TUM	Meningioma	No 5-ALA	2.0	0.1
3	55	m	TUM	Meningioma	+	11.7	0.5
4	73	m	INF	Atypical meningioma WHO II	-	2.5	0.6
5	59	f	TUM	Meningothelial meningioma WHO I	No 5-ALA	2.9	0.2
6	79	f	TUM	Meningothelial meningioma WHO I	+	8.6	2.2
7	41	m	TUM	Meningothelial meningioma WHO I	No 5-ALA	2.4	0.3
8	65	f	TUM	Microcystic meningioma WHO I	No 5-ALA	3.0	0.7
9	55	m	FAT	Meningothelial meningioma WHO I	No 5-ALA	3.3	0.4
10	75	f	TUM	Transitional meningioma WHO I	+	15.0	0.2
11	59	f	TUM	Meningothelial meningioma WHO I	No 5-ALA	3.8	0.4
12	49	m	TUM	Transitional meningioma WHO I	No 5-ALA	2.2	0.4
13	69	f	TUM	Meningothelial meningioma WHO I	+	11.2	2.0
14	71	f	TUM	Transitional meningioma WHO I	No 5-ALA	1.6	0.2

15	48	f	INF	Atypical meningioma WHO II	+	11.2	1.1
16	56	m	TUM	Atypical meningioma WHO II	No 5-ALA	2.6	0.4
17	53	f	TUM	Meningothelial meningioma WHO I	+	9.8	1.8
18	77	m	TUM	Microcystic meningioma WHO I	+	10.4	2.5
19	61	f	TUM	Sectretory meningioma WHO I	-	4.2	0.9
20	28	m	TUM	Atypical meningioma WHO II	+	14.2	0.3
21	52	f	TUM	Meningothelial meningioma WHO I	No 5-ALA	3.5	0.5
22	80	m	TUM	Fibrous meningioma WHO I	+	13.2	0.2
23	76	f	TUM	Atypical meningioma WHO II	No 5-ALA	2.1	0.2
24	72	m	TUM	Meningothelial meningioma WHO I	No 5-ALA	2.9	0.6
25	86	f	TUM	Meningothelial meningioma WHO I	+	9.5	0.8
26	75	f	TUM	Sectretory meningioma WHO I	No 5-ALA	2.2	0.7
27	64	f	TUM	Fibrous meningioma WHO I	+	13.8	0.1
28	53	f	INF	Meningothelial meningioma WHO I	-	4.9	0.9
29	82	f	TUM	Psammomatous meningioma WHO I	No 5-ALA	3.2	0.2
30	69	f	TUM	Meningothelial meningioma WHO I	No 5-ALA	2.0	0.4
31	68	f	TUM	Meningothelial meningioma WHO I	No 5-ALA	2.2	0.5
32	60	f	TUM	Atypical meningioma WHO I-II	+	13.3	0.8
33	68	f	TUM	Atypical meningioma WHO II	No 5-ALA	3.2	0.2

34	52	m	TUM	Atypical meningioma WHO II	No 5-ALA	2.3	0.5
35	66	m	TUM	Anaplasatic meningioma WHO III	+	14.4	0.7
36	70	m	TUM	Meningothelial meningioma WHO I	No 5-ALA	1.8	0.6
37	39	m	TUM	Meningothelial meningioma WHO I	+	13.2	1.6
38	47	f	TUM	Transitional meningioma WHO I	No 5-ALA	2.5	0.3
39	49	f	TUM	Transitional meningioma WHO I	No 5-ALA	2.2	0.5
40	55	f	TUM	Microcystic/ choroid meningioma WHO II	No 5-ALA	2.2	0.7
41	79	m	TUM	Transitional meningioma WHO I	+	12.8	0.9
**Total**	**N patients = 41, N specimens = 41**						
